# A Risk Prediction Model for the Assessment and Triage of Women with Hypertensive Disorders of Pregnancy in Low-Resourced Settings: The miniPIERS (Pre-eclampsia Integrated Estimate of RiSk) Multi-country Prospective Cohort Study

**DOI:** 10.1371/journal.pmed.1001589

**Published:** 2014-01-21

**Authors:** Beth A. Payne, Jennifer A. Hutcheon, J. Mark Ansermino, David R. Hall, Zulfiqar A. Bhutta, Shereen Z. Bhutta, Christine Biryabarema, William A. Grobman, Henk Groen, Farizah Haniff, Jing Li, Laura A. Magee, Mario Merialdi, Annettee Nakimuli, Ziguang Qu, Rozina Sikandar, Nelson Sass, Diane Sawchuck, D. Wilhelm Steyn, Mariana Widmer, Jian Zhou, Peter von Dadelszen, for the miniPIERS Study Working Group

**Affiliations:** 1 Department of Obstetrics and Gynaecology and the CFRI Reproductive and Healthy Pregnancy Cluster, University of British Columbia, Vancouver, Canada; 2 School of Population and Public Health, University of British Columbia, Vancouver, Canada; 3 Department of Anesthesiology, Pharmacology and Therapeutics, University of British Columbia, Vancouver, Canada; 4 Department of Obstetrics and Gynaecology, Stellenbosch University and Tygerberg Hospital, Cape Town, South Africa; 5 Centre of Excellence Division of Women and Child Health, Aga Khan University, Karachi, Pakistan; 6 Jinnah Post-graduate Medical College, Karachi, Pakistan; 7 Department of Obstetrics and Gynecology, Makerere University, Kampala, Uganda; 8 Department of Obstetrics and Gynecology, Feinberg School of Medicine Northwestern University, Chicago, Illinois, United States of America; 9 Department of Epidemiology, University Medical Center Groningen, University of Groningen, Groningen, The Netherlands; 10 Department of Obstetrics and Gynaecology, Colonial War Memorial Hospital, Suva, Fiji; 11 Department of Medicine, University of British Columbia, Vancouver, Canada; 12 UNDP/UNFPA/WHO/World Bank Special Programme of Research, Development and Training in Human Reproduction (HRP), Department of Reproductive Health and Research (RHR), Geneva, Switzerland; 13 Division of Women and Child Health, Aga Khan University, Karachi, Pakistan; 14 Department of Obstetrics and Gynaecology, Universidade Federal de São Paulo, Maternidade de Vila Nova Cachoeirinha, São Paulo, Brazil; 15 Department of Obstetrics, Tongji University, Shanghai, P.R. China; Maternal Reproductive & Child Health, London School of Hygiene & Tropical Medicine, United Kingdom

## Abstract

**Background:**

Pre-eclampsia/eclampsia are leading causes of maternal mortality and morbidity, particularly in low- and middle- income countries (LMICs). We developed the miniPIERS risk prediction model to provide a simple, evidence-based tool to identify pregnant women in LMICs at increased risk of death or major hypertensive-related complications.

**Methods and Findings:**

From 1 July 2008 to 31 March 2012, in five LMICs, data were collected prospectively on 2,081 women with any hypertensive disorder of pregnancy admitted to a participating centre. Candidate predictors collected within 24 hours of admission were entered into a step-wise backward elimination logistic regression model to predict a composite adverse maternal outcome within 48 hours of admission. Model internal validation was accomplished by bootstrapping and external validation was completed using data from 1,300 women in the Pre-eclampsia Integrated Estimate of RiSk (fullPIERS) dataset. Predictive performance was assessed for calibration, discrimination, and stratification capacity. The final miniPIERS model included: parity (nulliparous versus multiparous); gestational age on admission; headache/visual disturbances; chest pain/dyspnoea; vaginal bleeding with abdominal pain; systolic blood pressure; and dipstick proteinuria. The miniPIERS model was well-calibrated and had an area under the receiver operating characteristic curve (AUC ROC) of 0.768 (95% CI 0.735–0.801) with an average optimism of 0.037. External validation AUC ROC was 0.713 (95% CI 0.658–0.768). A predicted probability ≥25% to define a positive test classified women with 85.5% accuracy. Limitations of this study include the composite outcome and the broad inclusion criteria of any hypertensive disorder of pregnancy. This broad approach was used to optimize model generalizability.

**Conclusions:**

The miniPIERS model shows reasonable ability to identify women at increased risk of adverse maternal outcomes associated with the hypertensive disorders of pregnancy. It could be used in LMICs to identify women who would benefit most from interventions such as magnesium sulphate, antihypertensives, or transportation to a higher level of care.

*Please see later in the article for the Editors' Summary*

## Abstract 2

Beth Payne and colleagues use a risk prediction model, the Pre-eclampsia Integrated Estimate of RiSk (miniPIERS) to help inform the clinical assessment and triage of women with hypertensive disorders of pregnancy in low-resourced settings.

*Please see later in the article for the Editors' Summary*

## Editors' Summary

**Background:**

Each year, ten million women develop pre-eclampsia or a related hypertensive (high blood pressure) disorder of pregnancy and 76,000 women die as a result. Globally, hypertensive disorders of pregnancy cause around 12% of maternal deaths—deaths of women during or shortly after pregnancy. The mildest of these disorders is gestational hypertension, high blood pressure that develops after 20 weeks of pregnancy. Gestational hypertension does not usually harm the mother or her unborn child and resolves after delivery but up to a quarter of women with this condition develop pre-eclampsia, a combination of hypertension and protein in the urine (proteinuria). Women with mild pre-eclampsia may not have any symptoms—the condition is detected during antenatal checks—but more severe pre-eclampsia can cause headaches, blurred vision, and other symptoms, and can lead to eclampsia (fits), multiple organ failure, and death of the mother and/or her baby. The only “cure” for pre-eclampsia is to deliver the baby as soon as possible but women are sometimes given antihypertensive drugs to lower their blood pressure or magnesium sulfate to prevent seizures.

**Why Was This Study Done?:**

Women in low- and middle-income countries (LMICs) are more likely to develop complications of pre-eclampsia than women in high-income countries and most of the deaths associated with hypertensive disorders of pregnancy occur in LMICs. The high burden of illness and death in LMICs is thought to be primarily due to delays in triage (the identification of women who are or may become severely ill and who need specialist care) and delays in transporting these women to facilities where they can receive appropriate care. Because there is a shortage of health care workers who are adequately trained in the triage of suspected cases of hypertensive disorders of pregnancy in many LMICs, one way to improve the situation might be to design a simple tool to identify women at increased risk of complications or death from hypertensive disorders of pregnancy. Here, the researchers develop miniPIERS (Pre-eclampsia Integrated Estimate of RiSk), a clinical risk prediction model for adverse outcomes among women with hypertensive disorders of pregnancy suitable for use in community and primary health care facilities in LMICs.

**What Did the Researchers Do and Find?:**

The researchers used data on candidate predictors of outcome that are easy to collect and/or measure in all health care settings and that are associated with pre-eclampsia from women admitted with any hypertensive disorder of pregnancy to participating centers in five LMICs to build a model to predict death or a serious complication such as organ damage within 48 hours of admission. The miniPIERS model included parity (whether the woman had been pregnant before), gestational age (length of pregnancy), headache/visual disturbances, chest pain/shortness of breath, vaginal bleeding with abdominal pain, systolic blood pressure, and proteinuria detected using a dipstick. The model was well-calibrated (the predicted risk of adverse outcomes agreed with the observed risk of adverse outcomes among the study participants), it had a good discriminatory ability (it could separate women who had a an adverse outcome from those who did not), and it designated women as being at high risk (25% or greater probability of an adverse outcome) with an accuracy of 85.5%. Importantly, external validation using data collected in fullPIERS, a study that developed a more complex clinical prediction model based on data from women attending tertiary hospitals in high-income countries, confirmed the predictive performance of miniPIERS.

**What Do These Findings Mean?:**

These findings indicate that the miniPIERS model performs reasonably well as a tool to identify women at increased risk of adverse maternal outcomes associated with hypertensive disorders of pregnancy. Because miniPIERS only includes simple-to-measure personal characteristics, symptoms, and signs, it could potentially be used in resource-constrained settings to identify the women who would benefit most from interventions such as transportation to a higher level of care. However, further external validation of miniPIERS is needed using data collected from women living in LMICs before the model can be used during routine antenatal care. Moreover, the value of miniPIERS needs to be confirmed in implementation projects that examine whether its potential translates into clinical improvements. For now, though, the model could provide the basis for an education program to increase the knowledge of women, families, and community health care workers in LMICs about the signs and symptoms of hypertensive disorders of pregnancy.

**Additional Information:**

Please access these websites via the online version of this summary at http://dx.doi.org/10.1371/journal.pmed.1001589.

- The World Health Organization provides guidelines for the management of hypertensive disorders of pregnancy in low-resourced settings
- The Maternal and Child Health Integrated Program provides information on pre-eclampsia and eclampsia targeted to low-resourced settings along with a tool-kit for LMIC providers
- The US National Heart, Lung, and Blood Institute provides information about high blood pressure in pregnancy and a guide to lowering blood pressure in pregnancy
- The UK National Health Service Choices website provides information about pre-eclampsia
- The US not-for profit organization Preeclampsia Foundation provides information about all aspects of pre-eclampsia; its website includes some personal stories
- The UK charity Healthtalkonline also provides personal stories about hypertensive disorders of pregnancy
- MedlinePlus provides links to further information about high blood pressure and pregnancy (in English and Spanish); the MedlinePlus Encyclopedia has a video about pre-eclampsia (also in English and Spanish)
- More information about miniPIERS and about fullPIERS is available

## Introduction

The hypertensive disorders of pregnancy (HDP), and in particular pre-eclampsia and eclampsia, remain one of the top three causes of maternal mortality and morbidity, globally [1]–[4]. Pre-eclampsia also increases fetal risks, having been found to be associated with increased risk of stillbirth, neonatal death, intrauterine growth restriction, and preterm birth [4]. The majority of deaths associated with HDP occur in the low- and middle- income countries (LMICs) in the absence of a trained health professional [5],[6]. The increased burden of adverse outcomes in LMICs is believed to be due primarily to delays in triage (identification of who is, or may become, severely ill and should seek a higher level of care), transport (getting women to appropriate care), and treatment (provision of appropriate treatment such as magnesium sulphate, antihypertensives, and timed delivery) [7]–[9]. A major contributing factor to the morbidity and mortality associated with pre-eclampsia is the shortage of health workers adequately trained in the detection and triage of suspected cases [9].

One method suggested for enhancing outcomes in LMICs is task-shifting aspects of antenatal care to existing cadres of mid-level health workers [5],[10]. To do this effectively, these health workers require simple, evidence-based tools for monitoring pregnant women and accurately identifying who is at greatest risk of severe complications. By identifying those women at highest risk of adverse maternal outcomes well before that outcome occurs, transportation and treatment can be targeted to those women most in need.

Our group has previously developed the Pre-eclampsia Integrated Estimate of RiSk (fullPIERS) clinical prediction model, which predicts adverse maternal outcomes among women with pre-eclampsia on the basis of a woman's gestational age at diagnosis, the symptom complex of chest pain and/or dyspnoea, oxygen saturation by pulse oximetry, and laboratory results of platelet count, serum creatinine, and aspartate transaminase. The fullPIERS model, validated in a high-income tertiary hospital setting, has excellent discriminatory ability with an area under the receiver operating characteristic curve (AUC ROC) of 0·88 (95% CI 0·84–0·92) [11]. However, due to the inclusion of laboratory tests, the fullPIERS model may not be suitable for all settings, particularly primary care settings in LMICs.

The objective of the miniPIERS study was to develop and validate a simplified clinical prediction model for adverse maternal outcomes among women with HDP for use in community and primary health care facilities in LMICs.

## Methods

### Study Design and Population

The miniPIERS model was developed and validated on a prospective, multicentre cohort of women admitted to a participating centre with an HDP. Participating institutions were: the Colonial War Memorial Hospital, Suva, Fiji; Mulago Hospital, Kampala, Uganda; Tygerberg Hospital, Cape Town, South Africa; Maternidade Escola de Vila Nova Cachoeirinha, São Paulo, Brazil; Aga Khan University Hospital and its secondary level hospitals at Garden, Karimabad and Kharadar and Jinnah Post-graduate Medical College, Karachi, Pakistan; and Aga Khan Maternity & Child Care Centre, and Liaqat University of Medical Sciences, Hyderabad, Pakistan. Ethics approval for this study was obtained from each participating institution's research ethics board as well as the clinical research ethics board at the University of British Columbia. All participating institutions had a hospital policy of expectant management for women with pre-eclampsia remote from term, and similar guidelines for treatment of women with regard to magnesium sulphate and antihypertensive agents. Institutions were chosen to participate on the basis of the consistency of these guidelines in order to achieve some level of homogeneity within the cohort and to reduce systematic bias that could result from differences in disease-modifying practices between institutions.

Women were admitted to the study with any HDP defined as follows: pre-eclampsia, defined as (i) blood pressure (BP) ≥140/90 mmHg (at least one component, twice, ≥4 and up to 24 hours apart, after 20 weeks) and either proteinuria (of ≥2+ by dipstick, ≥300 mg/d by 24 hour collection, or ≥30 g/mol by urinary protein:creatinine ratio) or hyperuricaemia (greater than local upper limit of local non-pregnancy normal range); (ii) haemolysis, elevated liver enzymes, and low platelets (HELLP) syndrome even in the absence of hypertension or proteinuria [1]; or (iii) superimposed pre-eclampsia (clinician-defined rapid increase in requirement for antihypertensives, systolic BP [sBP] ≥170 mmHg or diastolic BP [dBP] ≥120 mmHg, new proteinuria, or new hyperuricaemia in a woman with chronic hypertension); or an “other” HDP defined as: (i) gestational hypertension (BP≥140/90 mmHg [at least one component, twice, ≥4 hours apart, ≥20^+0^ weeks] without significant proteinuria); (ii) chronic hypertension (BP≥140/90 mmHg before 20^+0^ weeks' gestation); or (iii) partial HELLP (i.e., haemolysis and low platelets OR low platelets and elevated liver enzymes). All women participating in the study gave informed consent according to local ethics board requirements.

Women were excluded from the study if they were admitted in spontaneous labour, experienced any component of the adverse maternal outcome before eligibility or collection of predictor variables, or had confirmed positive HIV/AIDS status with CD4 count <250 cells/ml or AIDS-defining illness.

Candidate predictor variables for final model development were identified *a priori* as being those variables that: (i) would be available and easy to collect in all health care settings including the woman's home; (ii) have been shown to be associated with pre-eclampsia in previous studies [12]; and (iii) would be measurable using simple and reliable methods. These variables included demographics (maternal age, parity, and gestational age on admission); symptoms (headache, visual disturbances, chest pain/dyspnoea, right upper quadrant pain or epigastric pain, nausea, vomiting, and vaginal bleeding with abdominal pain); and signs (blood pressure and dipstick proteinuria). The values for these variables were collected prospectively from the woman's medical record as measured by the nurse or physician during regular antenatal, intrapartum, or postnatal care. If multiple measures of a candidate predictor were collected within the first 24 hours of admission, the worst predictor value obtained within that first 24 hours of admission was used. The value used was the worst in the clinical context, this could either be the highest or lowest value collected in the given 24 hour time period, depending on the measure in question. This method of using the worst value was chosen as it is consistent with clinical practice. Generally, clinicians will respond to the worst clinical value when making management decisions.

The components of the composite adverse maternal outcome to be predicted by the model were determined by Delphi consensus [13] and include maternal mortality or one or more of serious central nervous system, cardiorespiratory, renal, hepatic, haematological, or other morbidity. The Delphi consensus process involved iterative review and feedback on the proposed outcome components from an expert group consisting of researchers and clinicians from both high- and low- or middle- income countries who have published work focused on HDPs. Representatives of the Delphi group brought expertise from medicine, obstetrics, pediatrics, anaesthesia, and critical care with sub-specialty expertise in maternal-fetal medicine, nephrology, haematology, and placental biology. Data were collected on the occurrence of all outcome components at any time during admission but for the purpose of the model, only those that occurred within 48 hours of admission were considered. All study sites were instructed to collect information on any “other” adverse events the woman experienced during pregnancy or immediately postpartum as part of the regular data collection process. This was done to ensure balanced reporting of events across all sites. Any reported “other” events were adjudicated by the study Working Group during regular meetings, at which time the decision was made whether to include the reported outcome as a study outcome, or not.

A full description of data collected can be found on the study website (http://pre-empt.cfri.ca/OBJECTIVES/miniPIERS/Reference.aspx) and definitions of the adverse maternal outcome components are provided in Table S1.

The external validation study was performed using data from the fullPIERS [11] dataset. The fullPIERS study was performed to develop and validate a prediction model for assessing risk in women with confirmed diagnoses of pre-eclampsia in high-resourced settings. This model includes gestational age at admission, chest pain/dyspnoea, oxygen saturation, platelet count, creatinine, and aspartate aminotransferase. Participating centres were tertiary academic hospitals located in Canada (six), the UK (two), New Zealand (one), and Australia (one). Only the fullPIERS data collected after 1 March 2008 were used for this study as this portion of the fullPIERS cohort was collected using the same protocol, inclusion and exclusion criteria, and data collection tools as later used for miniPIERS. Prior to this date, the fullPIERS cohort did not include abdominal pain, vaginal bleeding, or any headache.

Any researcher interested in accessing the miniPIERS or fullPIERS data can do so through the Pre-eclampsia CoLaboratory (http://pre-empt.cfri.ca/OBJECTIVES/CoLaboratory.aspx). A summarized version of the study dataset is also available as supplementary Dataset S1.

### Data Quality and Missing Data

Data for the miniPIERS dataset were collected prospectively using standardized data collection forms and protocols for all sites and entered into a customized Microsoft Access database. As part of the study protocol, women were required to have at least one measure of proteinuria, blood pressure, and symptoms during the first 24 hours of admission. All data were reviewed for quality and consistency. When questions arose regarding data, these data were confirmed by re-review of the primary health record. Random review of 10% of cases was performed during the first year of the study to ensure data validity within and between study sites.

The sample size required for model development was determined on the basis of the minimum standard of ten events per effective variable considered in the model according to the formula *N* = (*n*×10)/I where *N* is the sample size, *n* is the number of candidate predictor variables, and I is the estimated event rate in the population [14]. An estimated event rate of 15% based on our pilot data was used; for a model with 15 effective candidate predictor variables (i.e., dipstick proteinuria is counted three times to reflect inclusion of three indicator variables), the sample size required was 1,000 women [15],[16]. This sample size target was doubled to allow for subgroup analysis at the conclusion of the study after the finding of confounding by centre during the interim analysis.

### Statistical Methods

#### Coding of predictors

The relationship between each predictor variable and the combined adverse maternal outcome was first assessed by univariate logistic regression. Continuous variables were assessed for non-linearity, and were modeled as restricted cubic splines when appropriate [14]. Variables with a skewed distribution were log-transformed (natural log). Inclusion of the transformed variable in the final model was based on comparison to a model with the linear variable and selection of the model with the lowest Akaike information criterion (AIC) was automated during the model development process.

To avoid co-linearity, correlation between variables was determined and only the more clinically relevant variable of a pair of highly correlated variables was retained. When a high degree of correlation existed between two symptoms (r>0.5) they were re-coded as a combined indicator variable.

#### Model building

Stepwise backward elimination was used to build the most parsimonious model with a stopping rule of *p*<0·20. No interaction terms were included in the model as no interaction was hypothesized between candidate predictors prior to analysis.

We assessed the potential for confounding by study site by examining the bivariate association of study site with predictor variables and with outcome rate. Dummy (indicator) variables for study site were included in the model to eliminate confounding of the predictor-adverse outcome relationship by study site. To make the final model generalizable to all study settings, the coefficients for site variables were excluded from the calculation of predicted probability, and the model's intercept was adjusted using previously published methods for updating a prediction model for a new setting [14].

#### Assessing the model's performance

Calibration ability of the model was assessed visually by plotting deciles of predicted probability of an adverse maternal outcome against the observed rate in each decile and fitting a smooth line [14],[17]. Discrimination ability was evaluated on the basis of AUC ROC [18]. The sensitivity, specificity, positive predictive value, negative predictive value, and likelihood ratios (LRs) of cut-offs for a positive test defined using the population within each risk group were calculated [19]. The following categories for interpretation of the LRs were used: informative (LR<0·1 or >10); moderately informative (LR 0·1–0·2 or 5–10); and non-informative (LR 0·2–5).

A risk stratification table was generated to assess the extent to which the model's predictions divided the population into clinically distinct risk categories [20].

#### Model validation

Internal validation of the model was assessed using 500 iterations each of Efron's enhanced bootstrap method [21]. Details of this approach have been described previously [11],[14]. The bootstrapping procedure involved (i) sampling with replacement from the original cohort to generate a bootstrap dataset of 2,081 women; (ii) redevelopment of the model including all model development steps; variable coding (transformations and categorizations), variable selection, and parameter estimation in the bootstrapped sample; (iii) estimation of the AUC ROC for the model in the bootstrap sample; (iv) application of this new model to the original dataset and estimation of AUC ROC. Model optimism is then calculated as the average difference between model performance in the bootstrap sample and the original dataset after 500 iterations of this procedure. The choice was made to use 500 iterations because previous studies have shown no benefit is achieved when using a higher number of repetitions [16]. A final assessment of calibration was performed using the Hosmer-Lemeshow goodness-of-fit test.

A final assessment of model validity was performed by applying the miniPIERS model to the fullPIERS dataset and estimating the AUC ROC. Due to the marked difference in underlying rate of outcomes in the fullPIERS population (6.5% in fullPIERS versus 12.5% in miniPIERS), the model intercept (i.e., the baseline rate) was adjusted before estimating predictive performance [14]. This difference in outcome rate between the two cohorts is due to the difference in setting in which the data was collected, as noted in the description of the cohorts above, fullPIERS was completed in high-income country facilities only.

Sensitivity analyses were performed to assess the generalizability of the model in various subsets of study data. In addition, sensitivity analyses were performed excluding the most common components of the adverse maternal outcome to ensure that model discriminatory ability was maintained. Generalizability of the model across study regions was further assessed based on the AUC ROC calculated for the model when applied to each region's subset of the total miniPIERS cohort.

All statistical analyses were performed using STATA v11·0 (StataCorp).

## Results

From 1 July 2008 to 31 March 2012, 2,133 women were recruited to the miniPIERS cohort. Fifty-two of these women were excluded prior to analysis after review of their medical record revealed that they were ineligible. Medical chart review was able to resolve all instances of missing predictor variables in the total cohort. Data relating to the remaining 2,081 women were included in the model development and internal validation process. Compared with women who did not have an adverse outcome, women who had an adverse outcome were more likely to be nuliparous, to be admitted earlier in gestation, to be admitted with a diagnosis of pre-eclampsia, to have worse clinical measures in the first 24 hours of admission, and to have received corticosteroids and magnesium sulphate, but less likely to have been delivered by cesarean section (Table 1).

**Table 1:** Demographics of women in the total cohort comparing women with and without adverse maternal outcomes (*N* = 2,081).

Characteristic	Women with Adverse Outcomes (*n* = 401 women)	Women without Adverse Outcomes (*n = *1,680 women)	*p*-Value*
**Demographics (within 48 h of eligibility)**			
Maternal age at EDD (years)mean (±SD)	27·9 (±5·9)	28·5 (±6·2)	0·17
Parity ≥1*n* (%)	183 (45·6%)	939 (55·9%)	<0·01
Gestational age at eligibility (wk)median [interquartile range]	35·3 [30·7–38·1]	37·1 [34·1–38·8]	<0·01
Multiple pregnancy*n* (%)	17 (4·2%)	57 (3·4%)	0·41
Smoking in this pregnancy*n* (%)	25 (6·2%)	72 (4·3%)	0·08
**Pre-eclampsia description**			
*Pre-eclampsia* *n* (%)	320 (79·8%)	1016 (60·5%)	<0·01
*Other HDP* *n* (%)	81 (20·2%)	664 (39·5%)	<0·01
**Clinical measures (within 24 h of eligibility)**			
*Systolic BP*median [interquartile range]	170 [150–186]	150 [140–170]	<0·01
*Diastolic BP*median [interquartile range]	110 [100–120]	100 [90–110]	<0·01
Worst dipstick proteinuriamedian [interquartile range]	2+ [1+–3+]	1+ [trace–3+]	<0·01
Number of symptomsmedian [interquartile range]	1 [0–2]	0 [0–1]	<0·01
**Interventions at any time during admission**			
Corticosteroid administration*n* (%)	180 (44·9%)	525 (31·3%)	<0·01
Antihypertensive medications administered*n* (%)	386 (96·3%)	1,560 (92·9%)	0·13
MgSO_4_ administered*n* (%)	271 (67·6%)	677 (40·3%)	<0·01
**Pregnancy outcomes**			
Admission-to-delivery interval (all cases) (d)median [interquartile range]	1 [1]–[4]	1 [1]–[5]	0·02
GA on delivery (wk)median [interquartile range]	35·7 [31·7–38·3]	37·6 [35·3–39·1]	<0·01
Delivery at <34+0 wk GA*n* (%)	160 (39.9%)	290 (17.3%)	<0·01
Cesarean delivery*n* (%)	110 (27.4%)	625 (37.2%)	<0·01
Birth weight (g)median [interquartile range]	2,100 [1,303–2,800]	2,700 [2,000–3,150]	<0·01
Birth weight <3rd percentile (*N* babies)*n* (%)	64 (16·0%)	284 (16·9%)	0·66
Intrauterine fetal death(≥20+0 wk and/or ≥500 g)*n* (%)	54 (13·5%)	94 (5·6%)	<0·01
Neonatal death (before discharge)*n* (%)	26 (6·5%)	42 (2·5%)	<0·01

Results for continuous variables presented as mean (± standard deviation [SD]) when data normally distributed or median [interquartile range] for skewed data.

* *p*-Values calculated using chi-squared test for categorical variables and Student's t-test or Mann-Whitney U for continuous variables.

EDD, estimated date of delivery; GA, gestational age.

Maternal adverse outcomes included two maternal deaths during the study. The most common morbidities to occur were need for blood transfusion (174 women [8·4%]), placental abruption (70 women [3·4%]), and pulmonary oedema (51 women [2·5%]) (Table 2). There were 32 (1·5%) women with one or more seizures of eclampsia after admission, of whom 31 received magnesium sulphate.

**Table 2:** Maternal adverse outcomes occurring in the total miniPIERS cohort, outcome counts not mutually exclusive when listed within 48

One or More of Maternal Morbidity or Mortality:	Total Cohort (*N* = 2,081)	
	within 48 h	Any Time
**Total ** ***n*** ** (%)**	**261 (12·5%)**	**401 (19·3%)**
Maternal death	1	2
**Central nervous system**		
Eclampsia (≥1)	24	32
Glasgow coma score <13	8	11
Stroke or reversible ischaemic neurological deficit	3	4
Cortical blindness or retinal detachment	4	5
Posterior reversible encephalopathy	0	1
**Cardiorespiratory**		
Positive inotropic support	2	3
Infusion of a 3rd parenteral antihypertensive	8	9
Myocardial ischaemia/infarction	2	4
SpO_2_ <90%	9	22
≥50% FiO_2_ for >1 h	5	7
Intubation (other than for cesarean section)	14	25
Pulmonary oedema	37	51
**Haematological**		
Transfusion of any blood product	129	174
Platelets <50×10^9^/l with no transfusion	15	19
**Hepatic**		
Dysfunction	7	9
Haematoma/rupture	0	0
**Renal**		
Acute renal insufficiency	21	28
Dialysis	1	2
**Placental outcomes**		
Placental abruption	39	70
PPH requiring hysterectomy	39	50
**Other adverse events**		
Severe ascites	26	46
Other^a^	3	8

Full definitions of all outcomes available at http://pre-empt.cfri.ca/OBJECTIVES/miniPIERS/Reference.aspx.

a Includes five cases of pulmonary embolism, two cardiac arrests, one ruptured uterus.

SpO_2_, blood oxygen saturation; FiO_2_, fractional inspired oxygen; PPH, postpartum haemorrhage.

There was a strong correlation (r>0·5) between the symptoms of chest pain and dyspnoea, and headache and visual disturbances. Therefore, these symptoms were re-coded as combined indicator variables and entered accordingly into the multivariate model. As expected, systolic and diastolic blood pressure were highly correlated. Systolic blood pressure was selected for final model development because it is easier for minimally trained health care providers to measure by radial artery palpation than detection of Korotokoff sounds and it has been shown to be reflective of stroke risk in women with pre-eclampsia [22]. Systolic blood pressure measurements were log transformed for final model development as was gestational age at admission due to the highly skewed distribution of both variables.

Table 3 presents results of the univariate and multivariate analysis of miniPIERS predictors. The final miniPIERS equation was: logit (logarithm of the odds)(pi) = −5.77+[−2.98×10^−1^×indicator for multiparity]+[(−1.07)×log gestational age at admission]+[1·34×log systolic blood pressure]+[(−2·18×10^−1^)×indicator for 2 + dipstick proteinuria]+[(4·24×10^−1^)×indicator for 3 + dipstick proteinuria]+[(5.12×10^−1^)×indicator for 4 + dipstick proteinuria]+[1·18×indicator for occurrence of vaginal bleeding with abdominal pain]+[(4.22×10^−1^)×indicator for headache and/or visual changes]+[8.47×10^−1^×indicator for chest pain and/or dyspnoea].

**Table 3:** Univariate and multivariate analysis of candidate predictors in the miniPIERS cohort.

Candidate Predictor	Univariate OR [95% CI]	Multivariate OR [95% CI]
*Demographics*		
Maternal age (years)	0.99 [0.97–1.01]	n/a
Gestational age at admission (wk)	0.95 [0.92–0.98]	0.34 [0.11–1.11]^a^
Parity (multiparous versus primiparous)	0.73 [0.57–0.95]	0.74 [0.56–0.99]
*Signs*		
Systolic BP (mmHg)	1.02 [1.01–1.02]	3.89 [1.19–12.66]^a^
Diastolic BP (mmHg)	1.03 [1.02–1.03]	n/a
*Dipstick proteinuria*		
2+	1.44 [0.99–2.09]	0.80 [0.51–1.27]
3+	2.88 [2.07–4.00]	1.53 [0.99–2.37]
4+	3.23 [2.18–4.85]	1.67 [0.96–2.88]
*Symptoms*		
Headache	3.42 [2.58–4.52]	1.53 [1.07–2.17]
Visual disturbances	2.63 [2.00–3.45]	
Chest pain	6.42 [3.62–11.37]	2.33 [1.38–3.94]
Dyspnoea	6.35 [4.08–9.89]	
Epigastric/right upper quadrant pain	3.93 [2.96–5.21]	n/a
Nausea/vomiting	3.40 [2.53–4.57]	n/a
Abdominal pain with vaginal bleeding	6.03 [4.25–8.57]	3.24 [2.13–4.94]

Variables presented as part of the multivariate analysis are those that were retained after model development and backward selection.

a Log transformed.

OR, odds ratio.

**Figure 1:**
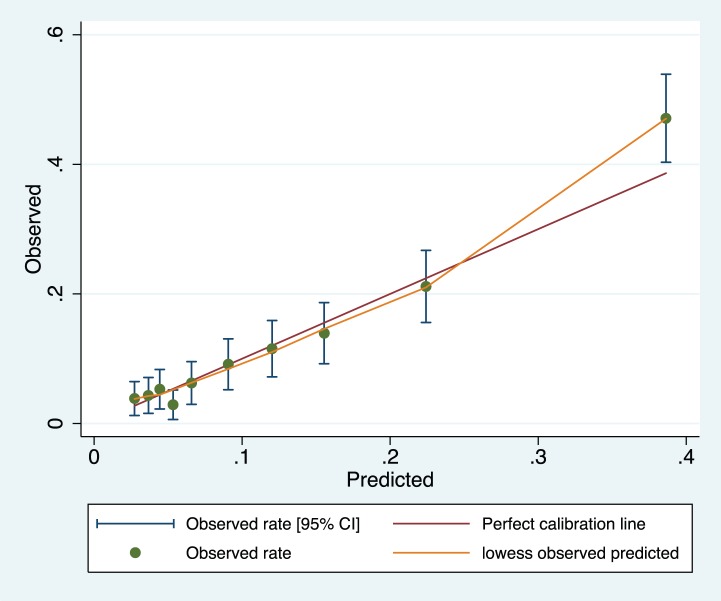
Calibration plot of the miniPIERS model applied 2,081 women in the cohort (H–L goodness of fit *p* = 0.1616). Green line represents line of perfect fit between observed and predicted outcomes and orange line is a smoothed fit line between predicted probability and mean observed probability in each range.

**Figure 2:**
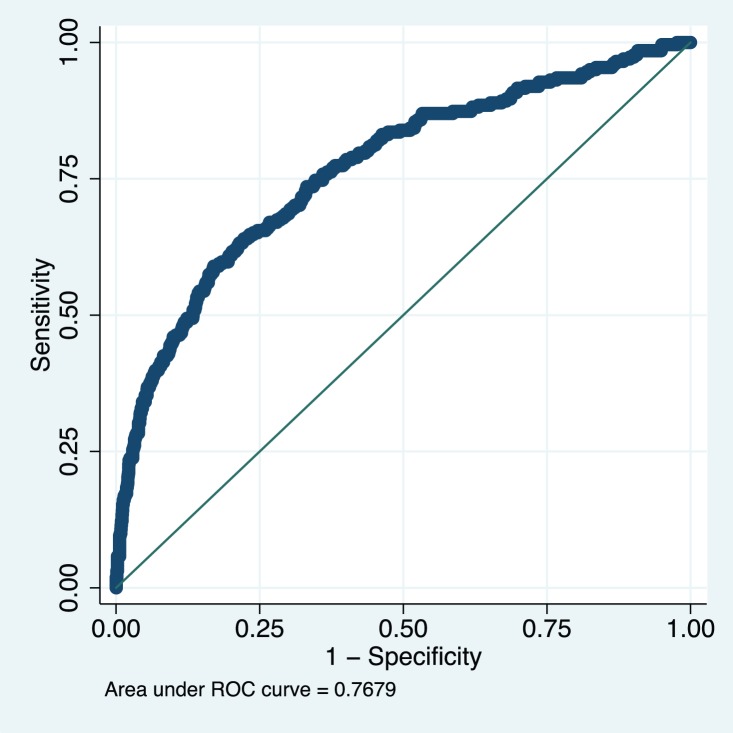
Receiver operating characteristic curve of the miniPIERS model developed in 2,081 women in the miniPIERS cohort. AUC 0.768 (95% CI 0.735–0.801).

The model appeared well-calibrated, as shown in the calibration plot (Figure 1). In all deciles except for the highest the 95% confidence interval around the observed outcome rate crossed the diagonal fitted line. The AUC ROC for this model was 0·768 (95% CI 0·735–0·801) (Figure 2) with an average optimism estimated to be 0.037. Using a cut-off of predicted probability of 25% to define a positive test resulted in a LR of 5.09 [4.12–6.29] and classified women with 85.5% accuracy (sensitivity 41.4%; specificity 91.9%). The stratification capacity of the model was good, as shown by the 784 (37.7%) and 256 (12.3%) women in the lowest and highest risk groups, respectively (Table 4).

**Table 4:** Risk stratification table to assess the miniPIERS prediction model.

Predicted Probability	*n* Event/*n* in Range	Percent Sens	Percent Spec	Percent PPV	Percent NPV	LR [95% CI]^a^
0–5·5%	33/784	—	—	—	—	0.31 [0.22–0.42]
5·6–8·0%	18/286	87.4	41.3	17.6	95.8	0.47 [0.29–0.74]
8·1–15·0%	46/456	80.5	56.0	20.8	95.2	0.78 [0.59–1.03]
15.1–24.9%	56/299	62.8	56.6	29.5	93.6	1.61 [1.24–2.08]
≥25%	108/256	41.4	91.9	42.2	91.6	5.09 [4.12–6.29]

Upper limit of predicted probability range used to define a positive test for sensitivity (Sens), specificity (Spec), positive predictive value (PPV), and negative predictive value (NPV).

a LR for each category calculated using the method described by Deeks et al. [19].

**Figure 3:**
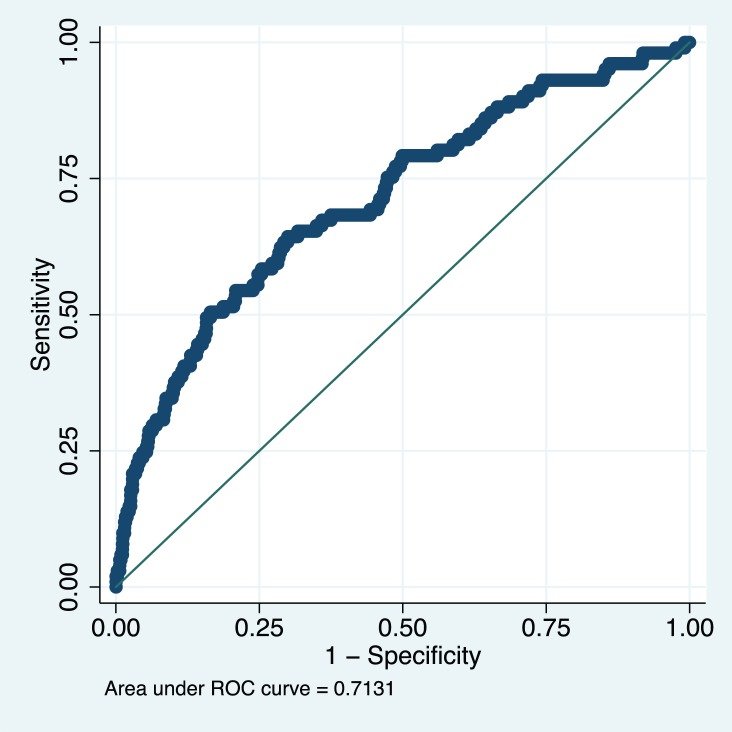
Receiver operating characteristic curve of the miniPIERS model applied to the fullPIERS (11) external validation cohort. AUC 0.713 (95% CI 0.658–0.768).

Data from 1,300 women in the fullPIERS cohort were used for external validation of the developed miniPIERS model. Table 5 presents the results of a comparison of demographics and clinical characteristics of women in fullPIERS compared to miniPIERS. The cohorts differed significantly with respect to demographics, interventions, and pregnancy outcomes. When the miniPIERS model was applied to the fullPIERS dataset the AUC ROC was 0.713 (95% CI 0.658–0.768) after adjusting the model intercept to account for differences in the outcome rate between the fullPIERS and miniPIERS populations (Figure 3).

**Table 5:** Demographic table comparing characteristics of women in the development and validation cohorts.

Characteristic	miniPIERS Cohort (*n* = 2,081 Women)	fullPIERS Cohort (*n* = 1,300 Women)	*p*-Value*
**Demographics (within 48 h of eligibility)**			
Maternal age at EDD (years)mean (±SD)	28.4 (±6.2)	31.7 (±6.0)	<0.01
Parity ≥1*n* (%)	1122 (53.9%)	403 (31.0%)	<0.01
Gestational age at eligibility (wk)median [interquartile range]	36.8 [33.5–38.7]	37.0 [34.1–38.9]	0.04
**Pre-eclampsia description**			
*Pre-eclampsia* *n (%)*	1,336 (64.2%)	1,020 (78.5%)	<0.01
*Other HDP* *n (%)*	745 (35.8%)	280 (21.5%)	<0.01
**Clinical measures (within 24 h of eligibility)**			
*Systolic BP*median [interquartile range]	160 [140–170]	166 [155–180]	<0.01
*Diastolic BP*median [interquartile range]	100 [95–110]	104 [98–110]	0.22
Worst dipstick proteinuriamedian [interquartile range]	2+ [trace–3+]	1+ [trace–3+]	0.01
Number of symptomsmedian [interquartile range]	1 [0–1]	1 [0–2]	<0.01
**Interventions at any time during admission**			
Corticosteroid administration*n* (%)	705 (33.9%)	337 (25.9%)	<0.01
Antihypertensive medications administered*n* (%)	1,946 (93.5%)	836 (64.3%)	<0.01
MgSO_4_ administered*n* (%)	948 (45.5%)	370 (28.5%)	<0.01
**Pregnancy outcomes**			
Admission-to-delivery interval (all cases) (d)median [interquartile range]	1 [1]–[4]	1 [1]–[4]	0.24
GA on delivery (wk)median [interquartile range]	37.3 [34.6–39.0]	37.6 [35.3–39.1]	0.16
Delivery at <34 + 0 wk GA*n* (%)	450 (21.6%)	319 (24.5%)	0.04
Adverse maternal outcome (within 48 h of admission)*n* (%)	261 (12.5%)	84 (6.5%)	<0.01
Birth weight (g)median [interquartile range]	2,600 [1900–3090]	2,836 [2105–3365]	<0.01
Intrauterine fetal death (≥20+0 wk and/or ≥500 g)*n* (%)	148 (7.1%)	15 (1.2%)	<0.01
Neonatal death (before discharge)*n* (%)	68 (3.3%)	14 (1.1%)	<0.01

Results for continuous variables presented as mean (± standard deviation [SD]) when data normally distributed or median (interquartile range) for skewed data.

* *p*-Values calculated using chi-squared test for categorical variables and Student's t-test or Mann-Whitney U for continuous variables.

EDD, estimated date of delivery; GA, gestational age.

The results of several sensitivity analyses done using the miniPIERS cohort are presented in Table 6. In all subsets, model performance was maintained. Of note, when the cohort was restricted to only those women admitted with a diagnosis of pre-eclampsia (defined as hypertension and proteinuria) the AUC ROC was 0.769 (0.733–0.807). In addition, when including the whole cohort but restricting the definition of the adverse outcome to include only maternal death, eclampsia, stroke, cortical blindness, or retinal detachment the AUC ROC was 0.811 (0.749–0.874). The model performance did not appear to differ significantly between study regions, although the confidence interval around the estimate of the AUC ROC in small study sites was wide (see Table 7).

**Table 6:** Results of sensitivity analysis using the miniPIERS model to predict adverse maternal outcome in subsets of the data or to predict restricted definition of the combined adverse outcome, as described, in the miniPIERS and fullPIERS cohorts.

Cohort Description	Outcome Incidence in miniPIERS Cohort (*n*/*N*)	AUC ROC [95% CI]	Outcome Incidence in fullPIERS Cohort (*n*/*N*)	AUC ROC [95% CI]
Including only women admitted with diagnosis of pre-eclampsia^a^	200/1,336	0.769 [0.733–0.807]	73/1,028	0.723 [0.647–0.793]
Including all but blood transfusion as adverse maternal outcome	174/2,081	0.762 [0.722–0.802]	68/1,300	0.758 [0.732–0.782]
Including all but PPH and placental abruption as adverse maternal outcome	240/2,081	0.776 [0.742–0.810]	n/a	n/a
Including maternal mortality, eclampsia, stroke, retinal detachment, or cortical blindness occurring at any time after admission only	38/2,081	0.811 [0.749–0.874]	n/a	n/a
Including only women admitted ≤34+6 wk GA	94/578	0.761 [0.703–0.818]	n/a	n/a
Including only women admitted >34+6 wk	167/1,503	0.767 [0.723–0.807]	49/973	0.729 [0.636–0.822]
Including only women admitted ≥37+0 wk GA	108/997	0.780 [0.731–0.829]	n/a	n/a

a Other hypertensive disorders excluded: chronic hypertension, gestational hypertension without proteinuria, or other adverse conditions, partial HELLP.

GA, gestational age.

**Table 7:** Performance of the model in each study site region as a predictor of combined adverse maternal outcome occurring within 48

Region	Contribution of Cases to Total miniPIERS Cohort (%)	Outcome Incidence in Cohort Used (n/N)	AUC ROC (95% CI)
Brazil	9.0	13/187	0.685 [0.524–0.826]
Fiji	6.1	5/127	0.721 [0.489–0.953]
Pakistan	50.7	157/1,056	0.758 [0.713–0.804]
South Africa	16.8	67/349	0.762 [0.702–0.821]
Uganda	17.4	19/362	0.656 [0.513–0.799]

Table 6 also presents sensitivity analyses performed using the fullPIERS cohort. Due to the smaller number of events in this cohort not all analyses could be meaningfully repeated but where performed, model performance appeared to be maintained.

## Discussion

Using data from a prospectively collected cohort of 2,081 women with HDP admitted to a hospital in five LMICs, we have developed and internally validated the miniPIERS model. The final miniPIERS model includes only demographics, symptoms, and signs that can be measured in primary health care facilities in low-resourced settings. Data for the study were collected by nurses and research staff with basic training to ensure the feasibility of replication of the measurements by comparable workers. For example, gestational age can be estimated from clinical information when ultrasound in unavailable, symptoms can be ascertained with simple questions, systolic blood pressure can be estimated easily using the radial pulse, and dipstick proteinuria can be estimated by assessing the opacity of boiled urine when dipsticks are not available [23]. By confining ourselves to these simple measures, the miniPIERS model has potential for use by mid-level health workers in low-resourced settings. To add to the ease of use of this model, miniPIERS is being converted to a mobile health application that will be useable on any mobile device so that health care workers are not required to calculate risk directly.

Overall, the miniPIERS model performed well on the basis of accuracy and discrimination ability (i.e., the AUC ROC). There was a slight underestimation of risk in the highest decile of predicted probability, but because the model was designed to be used as a categorical decision rule, this error in calibration is not thought to be clinically relevant. This model attains similar stratification, calibration, and classification accuracy as other established risk scores used in adult and reproductive medicine [24],[25]. To our knowledge, the miniPIERS model is the only clinical prediction model developed and validated for use with pregnant women in LMICs.

The miniPIERS model was used to designate women as being high-risk if their predicted probability of adverse outcome was ≥25%. The LR associated with this threshold showed potential utility as a rule-in test for adverse maternal outcome. By improving the ability of care providers to identify women at high risk of adverse outcomes, our specific aim was to reduce triage delays for women with any HDP in LMICs. What may be most useful is to set one threshold of predicted probability of adverse outcome, such as >15%, to initiate increased surveillance and use the higher threshold of ≥25% to initiate transport to a facility where emergency obstetric care is available. The positive predictive value of the 25% threshold was approximately 40% in all datasets with a corresponding 85% classification accuracy. These modest results highlight the fact that demographics, symptoms, and signs alone will not identify all women with severe disease but still have the potential to significantly improve care in resource limited areas and community settings where no or minimal monitoring of women with the HDP currently occurs.

There are several limitations to this study. The first is the use of a combined adverse maternal outcome comprising events of unequal severity. The Delphi consensus group determined that all components of the outcome were important enough on their own to warrant avoidance. The sensitivity analyses performed using a restricted definition of the adverse maternal outcome demonstrated that the model maintained its performance even when the more common and less-severe outcomes were excluded. A second limitation of the study is the use of broad inclusion criteria that included women with any HDP. This decision was made to make the model maximally useful for women who present with HDP, and for whom the exact diagnosis may not (or cannot) be determined at the time of clinical presentation. Reassuringly, when we restricted the cohort to only those women who were admitted with classically defined pre-eclampsia (hypertension and proteinuria), model performance was maintained.

A third limitation is the use of a backward elimination method for final variable selection in the model. Automated variable selection methods for model development have been shown to be sensitive to minor changes in the data and are not easily reproducible [26]. Ultimately, we felt that creating a simpler model with only those few variables that were most predictive of the outcome was important to make application of the model by minimally trained care providers easier.

A fourth limitation is the use of the fullPIERS dataset for external validation of the model. Although the data were collected for both fullPIERS and miniPIERS using the same definitions and protocols, the populations between the two studies differed significantly, as did the care received. Ideally the model should be validated in another cohort of data from low-resourced settings collected by mid-level care providers as part of routine care. This is planned and would address the possible concern for a reduction in model performance should these health workers be unable to maintain the level of measurement accuracy achieved in the facility data we have used for this study. In the interim, it was reassuring that there was consistency of results between fullPIERS and miniPIERS models. miniPIERS model performance was maintained in the fullPIERS cohort and more importantly coefficients were similar in overlapping predictors between the fullPIERS and miniPIERS models. This gives us confidence that this is a well-defined and stable model. A final limitation is the inclusion of clinically defined gestational age within the miniPIERS model, usually based on last menstrual period dates. As in fullPIERS, increasing gestational age was associated with diminishing risk [11]. This inverse relation was maintained in this study despite the inaccuracy inherent in clinically based gestational age assessment. Despite these limitations we were able to achieve accurate predictions from the miniPIERS model.

A major strength of this study is the high quality of data collected in a standardized manner. We were able to ensure that complete data were collected in five different LMICs through careful study monitoring and training of research staff. A second strength of this study is the generalizability of the resulting model. By combining high quality data from multiple international sites we are able to generate a model that should be applicable to any LMIC setting. The generalizability of the model is further supported by the results of the region-specific analysis of model performance. It is likely that we would have had greater predictive power had we developed the model using a more homogeneous population from one geographic region, but this would have resulted in a less generalizable model. By trading some predictive ability for generalizability, we believe we will have achieved greater impact on global public health. A final strength of the study is the use of clinically important timeframes for assessment and prediction. The miniPIERS model predicted adverse maternal outcomes occurring within 48 hours of assessment using data from within 24 hours of assessment; such timeframes represent clinically useful time periods in which transportation or disease-modifying interventions such as magnesium sulphate, antihypertensive agents, and delivery can be initiated.

When Thaddeus and Maine first proposed the three delay framework for explaining maternal mortality, they characterised the first delay as a “delay in deciding to seek care on the part of the individual, the family, or both” [9]. Factors that have been identified to influence this decision are the mother's level of education and health knowledge, perceived severity of the complication that is occurring, antenatal care attendance, and distance to facility [8],[9]. An additional barrier to women receiving quality care for HDP is the global crisis for human resources for health [6]. We believe that the miniPIERS model represents a significant step towards overcoming many of these barriers by providing evidence-based information on disease severity and allowing task-shifting of monitoring for complications related to HDP to mid-level health workers.

The potential implications of introduction of this model into routine antenatal care for LMICs are 2-fold: first, at the individual level women would not suffer the cost and time away from their families for unnecessary referrals when safe, increased community surveillance would be appropriate. Secondly, at the health systems level, evidence-based monitoring and primary triage for HDPs (especially pre-eclampsia) is moved from the tertiary facilities alone into lower level or primary health clinics, thereby increasing the potential for broad population-based screening, as well as making more efficient use of already burdened acute care facilities.

We believe that this clinical prediction tool is an important contribution as it offers the potential to improve health outcomes of women for a condition that is at the root of a large amount of morbidity and mortality in the developing world. Nevertheless, as with any prediction model, its ultimate value will only be demonstrated with an implementation project that is able to demonstrate that its potential can be translated to real health systems change and clinical improvements; such a project, called the Community Level Interventions for Pre-eclampsia (CLIP) study (clinicaltrials.gov ID NCT01911494), is presently underway. For more information on the CLIP study please see http://pre-empt.cfri.ca/OBJECTIVES/CLIPTrial.aspx). Until that study is complete, the miniPIERS model can be used as a basis of a community education programme to increase women's, families', and community-based health workers' knowledge of warning symptoms and signs associated with the HDP.

## Supporting Information

Dataset S1 **Data file containing predicted probability calculated by the miniPIERS model and observed outcome for all cases in the miniPIERS and fullPIERS cohorts.**
(XLSX)

Table S1 **Table of full definitions of maternal adverse outcomes used in the miniPIERS study.**
(DOCX)

## Abbreviations

AUC ROC: area under the receiver operating characteristic curve
BP: blood pressure
HDP: hypertensive disorders of pregnancy
HELLP: haemolysis, elevated liver enzymes, and low platelets
LMIC: low- and middle- income country
LR: likelihood ratio
PIERS: Pre-eclampsia Integrated Estimate of RiSk
